# Myrtle Syrup Improves Proteinuria in Type 2 Diabetic Patients: A Randomized Double-blinded Placebo-controlled Clinical Trial: Myrtle Syrup Improves Proteinuria in Type 2 Diabetes

**DOI:** 10.31661/gmj.v14i.3712

**Published:** 2025-07-02

**Authors:** Mohammad Saleh Solgi, Mohsen Bahrami, Mehdi Salehi, Naser Saeidi, Amir Almasi-Hashiani, Seyed Amirhossein Latifi

**Affiliations:** ^1^ Traditional and Complementary Medicine Research Center, Arak University of Medical Sciences, Arak, Iran; ^2^ Department of Internal Medicine, School of Medicine, Arak University of Medical Sciences, Arak, Iran; ^3^ Department of Epidemiology, School of Health, Arak University of Medical Sciences, Arak, Iran; ^4^ Zanjan University of Medical Sciences, Zanjan, Iran

**Keywords:** Myrtle, Proteinuria, upplementation, Type 2 Diabetes Mellitus

## Abstract

**Background:**

The use of medicinal plants as an alternative to synthetic drugs is increasing due to their accessibility and safety. In Iranian traditional medicine, myrtle (Myrtus communis) is widely recommended for treating kidney diseases, but scientific evidence supporting this claim is lacking. This study aimed to investigate the therapeutic effect of myrtle syrup (M. syrup) on proteinuria in patients with type 2 diabetes.

**Materials and Methods:**

This randomized, double-blind, placebo-controlled trial included 62 subjects aged 18–75 years with type 2 diabetes. Participants were randomly assigned to receive either M. syrup (10 cc) twice daily or a placebo syrup for 24 days. Enzyme-based commercial kits were used to measure serum levels of hemoglobin A1c (HbA1c), fasting blood sugar (FBS), blood urea nitrogen (BUN), creatinine, and protein in both serum and urine. 24-hour urine volume was also measured. Data analysis was performed using likelihood ratio chi-square tests, with statistical significance set at P0.05.

**Results:**

The results showed that M. syrup significantly improved proteinuria compared with the placebo group (P0.001). The mean change in urine protein was a decrease of 129 units in the intervention group and an increase of 16.5 units in the placebo group. However, no significant effects were observed on FBS, HbA1c, BUN, urine volume, serum creatinine, and urine creatinine. The potential mechanism of action for M. syrup in reducing proteinuria may be attributed to its antioxidant and anti-inflammatory properties.

**Conclusion:**

M. syrup supplementation may be an effective adjunct therapy for proteinuria in patients with type 2 diabetes. Hence, this should be emphasized in this regard.

## Introduction

Among metabolic diseases, diabetes is the most important and serious disorder affecting humans [1]. According to information obtained from the International Diabetes Federation, the global prevalence of diabetes in 2011 was 366 million, and it is estimated to reach 552 million by 2030, with a relative prevalence of 7.7% [2]. Diabetes mellitus is one of the most important risk factors for certain disorders such as nephropathy, retinopathy, neuropathy, and cardiovascular diseases. Approximately 30% of patients with diabetes have diabetic nephropathy [3]. If diagnosis and treatment are not performed on time, it can lead to End-Stage Renal Disease (ESRD) [4].

Hyperlipidemia, hyperglycemia, high blood pressure, and obesity through oxidative stress and inflammation are the most important determining factors for the development of diabetic nephropathy, which causes damage to the glomerulus in many ways [5][6]. The most important urinary parameters for diagnosing diabetic nephropathy are albumin, creatinine, urea, total protein, transferrin, glomerular filtration rate (GFR), type 4 collagen, ceruloplasmin, tumor necrosis factor (TNF-α), interleukin 6 (IL-6), vascular endothelial growth factor (VEGF), beta 2 microglobulin (B2M) [7][8].

Among these parameters, the total urine protein measurement has better sensitivity and specificity for diagnosing and monitoring nephropathy [9]. Pharmacological interventions are essential for the treatment of diabetic nephropathy. On the other hand, the main focus of these studies is on the design of blood sugar and pressure-reducing agents.

Accordingly, blood sugar-lowering drugs, such as Metformin, Sulfonylurea, and Canagliflozin, and blood pressure-lowering drugs, such as angiotensin-converting enzyme (ACE) inhibitors, such as captopril, aldosterone antagonists, such as spironolactone, and angiotensin receptor blockers, such as losartan, are used in treating nephropathy [10][6]. However, these drugs are not definitive treatments and have various side effects such as dry cough, headaches, dizziness, itching, and fatigue [11][12], necessitating an urgent need for better treatment options.

Herbal medicine is one of the most common treatment methods in traditional medicine. Currently, many modern medical drugs are extracted from natural sources, many of which have roots in traditional medicine [13]. Due to problems with side effects and access to modern drugs, and on the other hand, due to obtaining favorable results in the case of using medicinal plants for patients, the desire to use herbal medicine has increased. Herbal medicines are known to exert their therapeutic effects through various mechanisms, including antioxidant, anti-inflammatory, immunomodulatory, and endothelium-protective effects. These mechanisms can effectively target the underlying pathophysiological processes involved in diabetic nephropathy, such as oxidative stress, inflammation, and endothelial dysfunction[14][15].

Despite the growing interest in herbal medicine, there is a lack of robust scientific evidence supporting their widespread use, particularly in the context of diabetic nephropathy. Challenges in conducting rigorous research on herbal medicines include standardization of extracts, quality control, and the need for well-designed clinical trials with adequate sample sizes and long-term follow-up. Therefore, the focus of today’s research is to obtain scientific evidence for the use of herbal medicines. Myrtus communis (M. communis) is a shrub and evergreen plant. The leaves of this aromatic plant have an invigorating smell similar to that of eucalyptus [16]. M. communis grew in high abundance from the northwest to the eastern Mediterranean region. M. communis is also of high economic importance due to the extraction of oil from its leaves and fruit [17], it is mentioned in Persian medicine sources that M. communis has a strengthening effect on the kidney and bladder.

In "Tab Akbari" the myrtle plant is used for kidney, liver, and bladder weakness, and nocturnal enuresis [18]. Also, in "Qarabadin Salehi" the use of myrtle in the form of paste and syrup is recommended to compensate for bladder and kidney weakness [19]. Additionally, Talebianpoor and Issa in separate studies showed that aqueous and alcoholic extracts of myrtle plants have strong antidiabetic effects [20]. Studies have also shown that M. communis has protective effects on the liver and blood pressure [21].

Several studies have shown that M. communis has antioxidative, anticancer, antibacterial, antifungal and antiviral [22]. It is hypothesized that M. communis may exert its beneficial effects on proteinuria through its antioxidant and anti-inflammatory properties, which can target the key pathophysiological processes involved in diabetic nephropathy. To the best of our knowledge, no previous study has investigated the effects of M. communis on proteinuria. Therefore, this study aimed to investigate the therapeutic effects of myrtle plants on proteinuria in patients with type 2 diabetes.

## Materials and Methods

**Figure-1:**
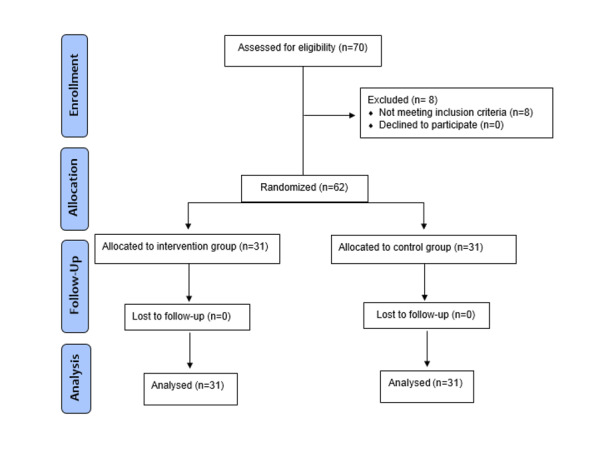
Figure 1. Summary of patient flow diagram

### Study Design

This study was a double-blind, randomized clinical trial to investigate the effect of M. syrup in diabetic patients with proteinuria. This study was approved by the ethics committee of Arak University of Medical Sciences (ethics code IR.ARAKMU.REC.1400.276) and registered in the Iranian Registry of Clinical Trials Center (registration no. IRCT20180610040049N8). After explaining the objectives of the study to the volunteers, signed informed consent was obtained from those willing to participate in the study.

### Study Participants and Sample Size

Seventy volunteers declared their readiness to participate in the study. However, based on the inclusion criteria, 62 patients were eligible to participate in the study. The inclusion criteria were as follows: type 2 diabetes with proteinuria, absence of serious heart disease (Participants with a history of serious heart conditions, such as heart attack or unstable angina, in the past 6 months were excluded from the study) in the last 6 months, age between 18 and 75 years, and absence of stage 3 or later kidney failure.

The Exclusion criteria were as follows: Occurrence of any adverse effects or complications related to the intervention or the underlying disease, non-compliance with the treatment regimen or study protocol, patient’s decision to withdraw from the study for any reason and loss to follow-up (Figure-1). The following formula (n=(Zα/2+Zβ)2 *2*σ2 / d2) was used to calculate the required sample size for each group, with a significance level of 5% and study power of 80%. The study by Saeedi et al. [23] estimated the standard deviation of 24-hour urine protein of diabetic patients to be approximately 67 mg, and to detect a difference of 50 units (approximately equal to one standard deviation) following the consumption of M. syrup, the minimum required sample size was estimated to be 28 subjects in each group. With the possibility of dropping out, 31 participants in each group were included in the study.

### Randomization and Allocation

To assign subjects to two groups (intervention and placebo), a block randomization method with block sizes of 4, 6, and 8 was used. It should be noted that concealment was also observed when using this method. In this method, each person was assigned a unique code and pasted it on the medicine package, which also helped the blinding process. The intervention group received M. syrup (10 cc) twice daily for 24 days, and the placebo group received placebo syrup, which was similar to M. syrup in terms of shape, size, color, smell, and packaging, twice daily for 24 days. Patients and data analysts were blinded to group type. Compliance was assessed through weekly phone follow-ups, during which participants were asked about their adherence to the treatment regimen and any difficulties they faced. Additionally, pill counts were performed at each follow-up visit to verify the number of syrup doses consumed. These measures provided a comprehensive assessment of participant compliance with the study protocol.

### Preparation of M. syrup and Placebo Syrup

The drug under study contained the aqueous extract of the common Myrtus plant in the pharmaceutical market of Iran with the scientific name M. communis. One hundred (100 g) of M. communis fruits were washed and soaked in 1000 cc of water in a beaker. After 3 h, the mixture was boiled for 10 min and cooled in a laboratory environment. A dry extract (8 g) was obtained from 100 g of M. communis fruit, and 5 g of the obtained extract was made up to 100 g using a USP syrup-making model with the amount of 66.7 grams of honey and 28.5 grams of water. The obtained syrup was poured into 120 cc sterile jars, sealed, and sterilized in an autoclave. The designed labels were then installed on them for clinical trials (USP 39-NF 34), Second Supplement Commentary, June 1, 2016). This syrup was made by the Traditional and Complementary Medicine Research Center, Arak University of Medical Sciences, Arak, Iran, by a botanist and traditional medicine expert.

### Assessment of Biochemical Variables

Biochemical variables were measured at the beginning and end of the study period. Enzyme-based commercial kits (Pars Azmoon, Tehran, Iran) with a spectrophotometer (Jenway 6505, Europe Union), according to the manufacturer’s instructions, were used to measure serum levels of hemoglobin A1c (HbA1c), urea, creatinine, albumin in serum and urine, and proteinuria.

### Statistical Analysis

Means and standard deviations were used to describe quantitative data and numbers and percentages were used as qualitative variables. Likelihood ratio chi-square analysis was used to compare qualitative variables. To compare the quantitative variables, the assumption of normal distribution of the data was evaluated using the Shapiro-Wilk test. However, since the assumption of normality was not established for the quantitative variables, the Mann-Whitney U test was used. To adjust the values ​​of the variables at the beginning of the study, the change score approach was used such that the mean difference of each variable in each group was calculated and compared between the two groups. All analyses were performed using Stata software version 13, and statistical significance was set at P<0.05.

## Results

**Table:** Table1. The Baseline Characteristics of Included Patients

**Variables **	**Intervention Group ** **n=31**	**Placebo Group ** **n=31**	**Total ** **n=62**	***P-value***
Age	51.8 (13.1)	56.9 (9.8)	54.4 (11.8)	0.872
Sex (2)	22 (71.0%)	23 (74.2%)	45 (72.6%)	0.776
Disease duration	5.6 (3.1)	5.2 (2.7)	5.4 (2.8)	0.632
BMI	25.6 (3.8)	25.3 (2.2)	25.4 (3.1)	0.650

As shown in Figure-1, 70 patients were evaluated according to the inclusion criteria, but eight patients were excluded from the study because they did not meet the criteria. Therefore, 62 patients were included in the study and were randomly divided into two intervention and control groups. No loss to follow-up was observed in any of the groups, and the patients were followed up until the end of the study. Ultimately, 31 patients from each group were included in the analysis.

### Demographics of the Study Participants

The basic data specifications of the study participants are listed in Table-1. The mean age of the participants in the study was 54.4 years (SD=11.8). About 45 cases (72.6%) of the participants had gender 2, the mean duration of the disease was 5.4 years (SD=2.8 years) and the mean BMI was 25.4 years (SD= 3.1). There were no significant differences between the two groups in terms of age (P=0.872), sex (P=0.776), disease duration (P=0.632), and BMI (P=650).

### The Outcome of M. communis Syrup on the Study Participants

The intended outcomes were compared between the two groups (Table-2). The mean of the desired indicators in both groups, before and after the study, and the difference between before and after were reported and compared. The comparison between the two groups was based on the observed mean differences. The pre- and post-intervention differences in each group were calculated and the mean differences between the groups were compared. Based on this, the mean changes observed in BUN between the two groups were not significant (P=0.490), while the changes in blood creatinine levels were significantly different between the two groups.

There was a decrease of 0.12 units in the intervention group and 0.02 units in the control group, respectively; these changes were statistically significant (P=0.012). The results of the analyses showed that the observed changes in HbA1c (P=0.333), FBS (P=0.750), urine creatinine (P=0.971), and urine volume (P=0.101) between the two groups were not significant, whereas the difference in the mean changes in urine protein between the intervention and placebo groups was significant (P=0.001).

In the intervention group, the mean urine protein level decreased by 129 units but increased by 16.5 in the placebo group. These changes were statistically significant between the two groups.

## Discussion

**Table:** Table2. Comparison of Interested Outcomes Between Two Groups of Intervention and Placebo

**Variables**		**Intervention Group ** **n=31**			**Placebo Group** **n=31**		***P-value*** **for difference **
	**Before**	**After**	**Mean Difference **	**Before**	**After**	**Mean Difference **	
BUN	16.5 (4.0)	16.6 (4.4)	0.07 (3.8)	17.6 (5.3)	18.2 (5.3)	0.66 (4.8)	0.409
Blood Creatinine	1.14 (0.25)	1.0 (0.24)	-0.12 (0.18)	1.0 (0.14)	0.97 (0.13)	-0.02 (0.09)	0.012
HbA1c	8.2 (1.5)	8.0 (1.4)	-0.19 (0.57)	7.8 (1.3)	7.8 (1.3)	-0.07 (0.32)	0.333
FBS	186.7 (66.9)	172 (61.4)	-14.5 (42.3)	187.7 (67.9)	172.2 (62.9)	-15.4 (39.7)	0.750
Urine Creatinine	1156.9 (447)	1125.4 (492)	-31.5 (380)	1123.0 (454)	1083.3 (411)	-39.7 (450)	0.971
Urine volume	1958.6 (786)	1683.1 (603)	-275.4 (665.9)	1563.6 (613)	1535.4 (661)	-28.3 (699)	0.101
Protein	328.8 (243)	199.1 (216.2)	-129.7 (192.7)	313.8 (249)	330.3 (256.9)	16.5 (95.6)	0.001

The prevalence of diabetes and diabetic nephropathy (DN) is increasing worldwide due to lifestyle changes. The use of medicinal plants for the treatment of metabolic diseases, including diabetes, DN, and fatty liver is increasing due to fewer side effects and availability for the treatment of these diseases. M. syrup is prescribed in Persian traditional medicine because of its beneficial effects in the case of diabetes and DN.

However, to date, no comprehensive study has investigated its effects. Hence, the effect of M. syrup on proteinuria in diabetic patients with DN was investigated for the first time in this study. The results of our study showed that consumption of M. syrup (10 cc) twice daily for 24 days significantly improved proteinuria and serum creatinine levels in patients. Additionally, the consumption of M. syrup (10 cc) twice daily for 24 days reduced HbA1c, and 24-hour urine volume compared to the placebo group, although the difference was not significant. This could be attributed to the relatively short intervention period (24 days) or the limited sample size. Longer-term studies with larger sample sizes may be needed to fully elucidate the effects of M. syrup on these parameters.

Additionally, the lack of significant changes in HbA1c and urine volume may suggest that the primary mechanism of action for M. syrup in improving proteinuria is not solely through glycemic control or diuretic effects. Further research is warranted to explore the specific mechanisms involved.

Nephropathy is one of the most important complications of diabetes. Proteinuria is an important biomarker for DN. Various mechanisms may lead to proteinuria [24]. Hemodynamic disturbances, including hyperfiltration and hypoperfusion, lead to albumin leakage into the Bowman's capsule [25]. In addition to these molecular mechanisms, oxidative stress and inflammatory processes, increases in prostanoids, nitric oxide (NO), atrial natriuretic factor (ANF), growth hormone, glucagon, insulin, angiotensin II (ANG II), accumulation of collagen 4 and fibrochitin, and damage to podocytes and others have been implicated as agents causing damage to the kidney structure and leading to proteinuria [26][27]. Ultimately, diabetes damages kidney structure and leads to proteinuria through oxidative stress and inflammatory processes. Therefore, treatment based on anti-inflammatory and antioxidant properties may be effective.

M. communis is used for the treatment of gastrointestinal, liver, and kidney diseases because of the successful treatment experiences reported in Persian medicine. It is affordable and readily available for consumption by the public. However, there is a lack of scientific evidence regarding its effect on the kidney, although useful biological effects, such as anti-herpes simplex virus type 1 activity [28], antioxidant [29], anti-inflammatory [30], anti-diarrheal [31], antiparasitic effects against Trichomonas vaginalis [32], and anti-respiratory infections [33] have been reported for M. communis. Ertas et al. reported that M. communis can improve ethylene glycol-induced nephropathy [34]. Interestingly, Rossi et al. reported that compounds in the leaves of M. communis can inhibit lipoxygenase and cyclooxygenase to prevent the formation of free radicals and inflammation. Additionally, Christian Feißt reported that myrtle plants have anti-inflammatory properties that prevent the mobilization of Ca2+ in polymorphonuclear leukocytes [30]. In a similar trajectory, Mustafaoğlu and her colleagues found that M. communis improved kidney and bladder damage in rats receiving a high-fat diet through the reduction of MDA, 8-OHdG, and MPO and an increase in GSH [35].

Studies have shown that the chemical composition of M. communis is mainly composed of phenolic compounds, including α-pinene, limonene, catechin, myricetin, myrtenal, and linalool [36][37]. The beneficial biological effects of M. communis are likely due to its chemical composition. Various studies on the effectiveness of M. communis compounds on diabetics have been conducted. In this regard, Babaeenezhad et al. reported that limonene could ameliorate gentamicin-induced nephropathy by suppressing the NF-κB Pathway, mitochondrial apoptosis, and oxidative stress [38]. Furthermore, Murali et al. reported that limonene had strong antidiabetic effects [39]. Zhu et al. also proposed that catechin may be a potential natural product as a metabolite of methylglyoxal (MG) scavenger against diabetes-related complications [40]. A study also suggested that myricetin alleviates renal tubular epithelial-mesenchymal transition via the NOX4/NF-κB/snail axis in diabetic nephropathy [41].

Similarly, Myrtenal has been reported to have antidiabetic and antihyperlipidemic effects in diabetic rats [42]. Linalool, another compound from M. communis, rescued the kidneys of diabetic rats from oxidative stress and inflammation by decreasing the expression of TGF-β1 and NF-κB [43]. Concurrent with our findings, these studies generated viable evidence that the chemical compounds of M. communis have anti-diabetic and anti-nephropathic effects. This study also demonstrated that M. syrup improved proteinuria and decreased urine creatinine levels in patients, suggesting that M. communis may have improved the structure of damaged nephrons.

The observed reduction in proteinuria and serum creatinine in the M. syrup group suggests potential improvements in renal structural or functional parameters. This could be attributed to various mechanisms, including: Improved glomerular filtration dynamics: M. syrup may enhance glomerular filtration by reducing inflammation and oxidative stress, leading to decreased leakage of protein into the urine. Podocyte protection: M. syrup may protect podocytes, specialized cells in the glomerulus that play a crucial role in preventing proteinuria, from damage caused by hyperglycemia and oxidative stress. Reduced tubular protein reabsorption: M. syrup may decrease protein reabsorption in the renal tubules, leading to increased excretion of protein in the urine. Further research is needed to investigate these potential mechanisms and confirm the direct impact of M. syrup on renal structure and function.

This study has several limitations, including: The 24-day intervention period may not be sufficient to fully assess the long-term effects of M. syrup on renal function, the limited sample size may have reduced the statistical power to detect significant differences in some outcomes and we did not analyze the specific active compounds in M. syrup, limiting our ability to determine the precise mechanisms of action. Despite these limitations, our study provides valuable preliminary evidence for the potential benefits of M. syrup in managing proteinuria in type 2 diabetes. Future studies with longer follow-up periods, larger sample sizes, and compound-specific analyses are needed to confirm and expand upon our findings.

## Conclusion

Traditional Persian medicine offers a good alternative to tackle chronic metabolic diseases, such as diabetes. In this pioneering clinical study, M. syrup consumption improved proteinuria in diabetic patients. Hence, it should be considered as an adjunct therapy for diabetic nephropathy. Nevertheless, we recommend further studies with larger sample sizes to ascertain the validity of our findings.

## Conflict of Interest

The authors declared that they have no conflict of interest.
